# Influence of muscle‐belly and tendon gearing on the energy cost of human walking

**DOI:** 10.1111/sms.14142

**Published:** 2022-02-14

**Authors:** Andrea Monte, Paolo Tecchio, Francesca Nardello, Beatriz Bachero‐Mena, Luca Paolo Ardigò, Paola Zamparo

**Affiliations:** ^1^ Department of Neurosciences, Biomedicine and Movement Sciences University of Verona Verona Italy; ^2^ Human Movement Science Faculty of Sports Science Ruhr University Bochum Bochum Germany; ^3^ Department of Physical Education and Sport Universidad de Sevilla Sevilla Spain

**Keywords:** belly gearing, cost of transport, human locomotion, pendular energy recovery

## Abstract

This study combines metabolic and kinematic measurements at the whole‐body level, with EMG and ultrasound measurements to investigate the influence of muscle‐tendon mechanical behavior on the energy cost (C_net_) of walking (from 2 to 8 km·h^−1^). Belly gearing (Gb = Δmuscle‐belly length/Δfascicles length) and tendon gearing (Gt = ∆muscle‐tendon unit length/∆muscle‐belly length) of vastus lateralis (VL) and gastrocnemius medialis (GM) were calculated based on ultrasound data. Pendular energy recovery (%R) was calculated based on kinematic data, whereas the cumulative activity per distance travelled (CMAPD) was calculated for the VL, GM, tibialis anterior, and biceps femoris as the ratio between their EMG activity and walking speed. Finally, total CAMPD (CMAPD_TOT_) was calculated as the sum of the CMAPD of all the investigate muscles. C_net_ and CMAPD_TOT_ showed a U‐shaped behavior with a minimum at 4.2 and 4.1 km·h^−1^, respectively; while %R, VL, and GM belly gearing showed an opposite trend, reaching a maximum (60% ± 5%, 1.1 ± 0.1 and 1.5 ± 0.1, respectively), between 4.7 and 5 km·h^−1^. Gt was unaffected by speed in GM (3.5 ± 0.1) and decreased as a function of it in VL. A multiple stepwise linear regression indicated that %R has the greatest influence on C_net,_ followed by CMAPD_TOT_ and GM belly gearing. The role of Gb on C_net_ could be attributed to its role in determining muscle work: when Gb increases, fascicles shortening decreases compared with that of the muscle‐belly, thereby reducing the energy cost of contraction.

## 1 INTRODUCTION

Walking is the most common locomotor task during daily life activities. It has been classically described by an inverted pendulum paradigm, where the potential and kinetic energies associated to the body center of mass (BCoM) are out of phase and continuously exchange (as in a pendulum‐like motion), reducing the total mechanical energy oscillations over a stride.^1^ In walking, the relationship between net energy cost (C_net_: the metabolic energy expended, above rest, to cover one unit distance) and speed can be empirically described by a quadratic (U‐shape) function that shows a minimum at a speed of about 4–5 km·h^−1^, often called the “optimal walking speed” which, in turn, is close to that of spontaneous walking.^2^

The U‐shape behavior of the C_net_ vs. velocity relationship could be associated with energy recovery behavior. Energy recovery is a parameter introduced by Cavagna et al. [1] to quantify the ability to save mechanical energy by using a pendulum‐like mechanism, whereas in an ideal pendulum, the energy exchange is complete (energy recovery is 100%), in walking energy recovery ranges between 40% and 70%, because of energy losses that are speed‐dependent^1^, ^2^; the pattern of pendular energy recovery as a function of walking speed shows an opposite trend compared with that of C_net_, reaching a maximum where the energy cost is minimized.^1^, ^2^, ^3^, ^4^

Pendular energy exchange can only occur within a single step: at the end of each step, the leading leg collides into the ground and negative work is performed, implying a loss of mechanical energy. In order to maintain steady walking speed (i.e., zero network over a stride), the lost energy should be replaced at the expense of muscular work.^5^, ^6^, ^7^ Moreover, muscular work is required to drive the “exchange” of kinetic and potential energy, particularly during the first half of the single support phase.^8^ Thus, in walking, the muscles perform external work to give an additional push forward during the middle of the double stance phase and to complete the lift during the middle of the single stance phase.^9^

The level of muscle force was generated to support and accelerate the body within the environment determines metabolic energy expenditure.^6^, ^7^, ^10^ This force, in turn, depends on the level of muscle activation and on the operating range of the muscle fascicles along with their force‐length (F‐L) and force‐velocity (F‐V) relationships.^11^, ^12^ During walking, plantar flexors muscles fascicles shorten during the entire stance phase^13^, ^14^, ^15^ and, when speed increases, the fascicles operating range and their shortening velocity increase too.^16^, ^17^ To maintain the requested level of force, more muscle fibers should be activated and/or the firing rate of the motor neuron should be increased. In both cases, this implies an increase in metabolic energy expenditure with increasing speed.^18^, ^19^ Therefore, the behavior of the plantar flexor muscles fascicles is in agreement with the increase in oxygen uptake as a function of speed but could not explain *per se* the U‐shape behavior of the C_net_ vs. velocity relationship in walking.

The other energy‐saving mechanism in terrestrial locomotion is related to the behavior of the elastic elements: tendinous tissues can store energy during the first half of the stance phase and return (part of) this energy in the later stance phase as a spring‐like bouncing mechanism.^3^, ^20^ Even if this mechanism plays a major role in running, tendons could store and release a significant amount of elastic energy during the stance phase of human walking too.^9^, ^13^, ^17^ However, the elastic energy released by the Achilles tendon remains stable as a function of walking speed^17^ and, therefore, the behavior of the Achilles tendon *per se* could not explain the U‐shape behavior of the C_net_ vs. velocity relationship either.

A new insight into the role of muscle and tendon behavior in determining the physiological responses of human walking can derive from the analysis of muscle‐belly and tendon gearing. When a pennate muscle contracts, its fibers can rotate and the pennation angle can thus increase. As a result, the shortening velocity of the muscle‐belly not necessarily equals that of its fibers.^21^ Muscle‐belly gearing (Gb = Δbelly length/Δfiber length) indicates the uncoupling behavior between whole‐muscle and fiber velocity^21^: a high belly gearing enables whole‐muscle shortening velocity to exceed fiber shortening velocity. Pennate muscles can vary belly gearing over their functional range to enhance shortening velocity without compromising force production.^21^, ^22^ Hence, the neuromuscular system can adapt its behavior to manipulate muscles’ mechanical behavior, following the request imposed by the task, affecting the metabolic cost of contraction (and therefore the energy cost of walking). In this regard, a geared muscle could operate with higher fascicles F‐L and F‐V potentials compared with a non‐geared muscle.^23^ Therefore, for a given walking speed, a neuromuscular system able to operate with higher values of belly gearing could result in a favorable condition to reduce the energy cost of walking.

Tendons, due to their compliance, could take over important portions of the length changes within the muscle‐tendon unit (MTU). This can substantially reduce the length change and velocity of the in‐series muscle‐belly. The uncoupling behavior between muscle‐belly velocity and MTU velocity (Gt = ΔMTU length/Δbelly length) has been called tendon gearing^22^, ^24^: for a given MTU length changes, the higher the tendon gearing the higher the MTU length changes accommodated by the elastic tissues (and the lower the muscle‐belly length changes). Therefore, a high tendon gearing allows the fascicles to operate under more isometric conditions, theoretically reducing the metabolic demands of a given locomotor task.^20^, ^24^, ^25^, ^26^ Taken together, these two mechanisms (tendon and belly gearing) could provide new insights into the physiological demands of human walking potentially helping to explain the changes in C_net_ as a function of speed (and the underpinning mechanisms of the optimal walking speed).

The aim of this study was to investigate (besides the role of energy recovery) the role of belly and tendon gearing of the lower limb muscles in determining the physiological response during walking at different speeds. To this aim, we combined EMG and ultrasound imaging of medial gastrocnemius and vastus lateralis muscle fascicles with a metabolic and kinematic analysis at the whole‐body level.

We expected to find that metabolic demands are primary related to pendular energy recovery and EMG activity of the investigated muscles (highest and lowest when C_net_ is minimized, respectively), and we hypothesized that belly gearing of the lower limb muscles will be the highest at the optimal walking speed, partially explaining the behavior of the energy cost of walking at increasing speed. Finally, we hypothesized a small/negligible influence of tendon gearing in determining the shape of the C_net_ vs. velocity relationship.

## 2 METHODS

### 2.1 Participants

Fourteen (7 males and 7 females) healthy subjects (age: 27.6 ± 4.6 years; body mass: 63.4 ± 11.6 kg; height: 1.69 ± 0.08 m; leg length: 81.1 ± 4.0 cm) participated in this study. All participants were in good health and reported no recent history of lower limb neuro‐musculoskeletal injury.

The study agreed with the Declaration of Helsinki for the study on human subjects. The local ethical committee approved the experimental protocol, and all subjects gave their written informed consent.

### 2.2 Data collection

Each subject participated in two experimental sessions. Before the first one, they familiarized with the equipment and procedures. Participants were asked to walk on a treadmill (H/P/Cosmos, Saturn 300/100r, Germany) and each testing condition (e.g., walking speed from 2 to 8 km·h^−1^, with 1 km·h^−1^ increments) was proposed in a random order using a self‐selected cadence and step length. The entire walking protocol was repeated twice in order to record ultrasound data of both vastus lateralis (VL) and gastrocnemius medialis (GM) with a randomized order. During the experiments, the EMG activity of the antagonist muscles (TA, tibialis anterior and BF, biceps femoris) was also recorded.

In the first session, the kinematics of the body segments, the EMG activity of the selected muscle (and of its antagonist) and its fascicle behavior were recorded along with metabolic data; in this case, the trials lasted 6 min with 10 min of passive recovery in‐between. In the second session, the participants walked for 1 min at each speed while kinematic, ultrasound, and EMG data of the other muscle (and of its antagonist) were recorded; in this second session, metabolic data were not recorded.

A 3D motion capture system (8 cameras; Vicon, Oxford, UK) was used to record the three‐dimensional trajectories of 49 markers (a customized full‐body Plug in Gait), sampling at 200 Hz.

During each trial, a B‐mode ultrasound scanner (Telemed MicrUs EXT‐1H rev. D, Lituania) was used to record images at 115 Hz with a depth and width of 40 and 60 mm, respectively. For both VL and GM, the probe was attached to the skin in the sagittal plane of the muscle‐belly, and adjusted until the image of the aponeuroses and fascicles were clearly visible, according to the recommendation of Van Hooren et al.^27^ The transducer laid fat on the skin and elastic bandages was tightly applied to minimize movement relative to the skin. The bandage completely covered the transducer, overlapping the edges minimizing any probe movements. To reduce the negative effect of muscle compression,^28^, ^29^ a soft gel pad was interposed between the probe and the skin. This procedure allows the muscles to expand radially.

The EMG signals of VL, GM, TA, and BF were collected using a wireless system (Aurion, Cometa, Italy) sampling at 1000 Hz. Ag‐AgCl bipolar electrodes were carefully placed over the muscle‐belly with an interelectrode distance of 2 cm after the skin surface was prepared by light abrasion and cleaned with an alcohol swab.^30^ Ultrasound, kinematic, and EMG data were synchronized by a digital output generated by the ultrasound scanner that triggered all instrumentation.

Oxygen uptake ($\dot{V}$O_2_) during each walking trial was determined by means of a breath by breath metabolimeter (K5, Cosmed, Italy). Six minutes of baseline values in the standing position were collected before the tests, and data were collected for six minutes during exercise. Data collected in the last minute of rest/exercise were averaged and used in further analysis.

### 2.3 Data analysis

Kinematic, EMG, and ultrasound data were analyzed for ten stance phases in the last minute of exercise; for each instrumentation data were interpolated to 200 sample points.

Marker trajectories were filtered with a forward and reverse 2^nd^ order, low pass Butterworth filter, with a cutoff frequency of 12 Hz. Inverse kinematics was used to calculate the angular rotation for each body segment. The 3D trajectory of the body center of mass (BCoM) was calculated as proposed by Minetti et al.^31^ based on the kinematics of 11 body segments. The mass and the radius of gyration of each segment were determined according to Dempster inertial parameters. Based on these data, the time course of potential and kinetic energies was computed and summed to calculate total mechanical energy over a stride (*ET*, expressed in J). The summation of all increases in *ET* time course constitutes the positive external work (W_ext_).^3^ Pendular energy recovery (%R) was calculated, for each trial, as follows:

$$\%R=\frac{\left|\left.\mathrm{Wv}\right|\right.+\left|\left.\mathrm{Wf}\right|\right.‐Wext}{\left|\left.\mathrm{Wv}\right|\right.+\left|\left.\mathrm{Wf}\right|\right.}x100$$

where Wv and Wf are the module of the vertical and forward mechanical work.^1^

Kinematic and ultrasound data were analyzed with a custom‐written software (LabVIEW 10, National Instrument, USA).

For the ultrasound measurements, muscle thickness, pennation angle, and fascicle length were post‐processed using a customized version of an automatic tracking algorithm proposed by.^32^, ^33^ At the end of the auto‐tracking, every frame of the tracked fascicle lengths was visually examined to check the algorithm accuracy. Whenever the fascicle length was deemed inaccurate, the two points defining the muscle fascicles were manually repositioned. The MTU length of VL and GM was computed, at each instant, using the instantaneous joint angles, as proposed by Hawkins and Hull.^34^ The length changes of the VL and GM muscle‐belly were calculated as the product of fascicle length and the respective cosine of the pennation angle. Note that this gives not the length of the entire muscle‐belly but the projection of the instant fascicle length to the plane of the MTU, which can be used to calculate the muscle‐belly length changes.^22^, ^24^, ^25^

**FIGURE 1:**
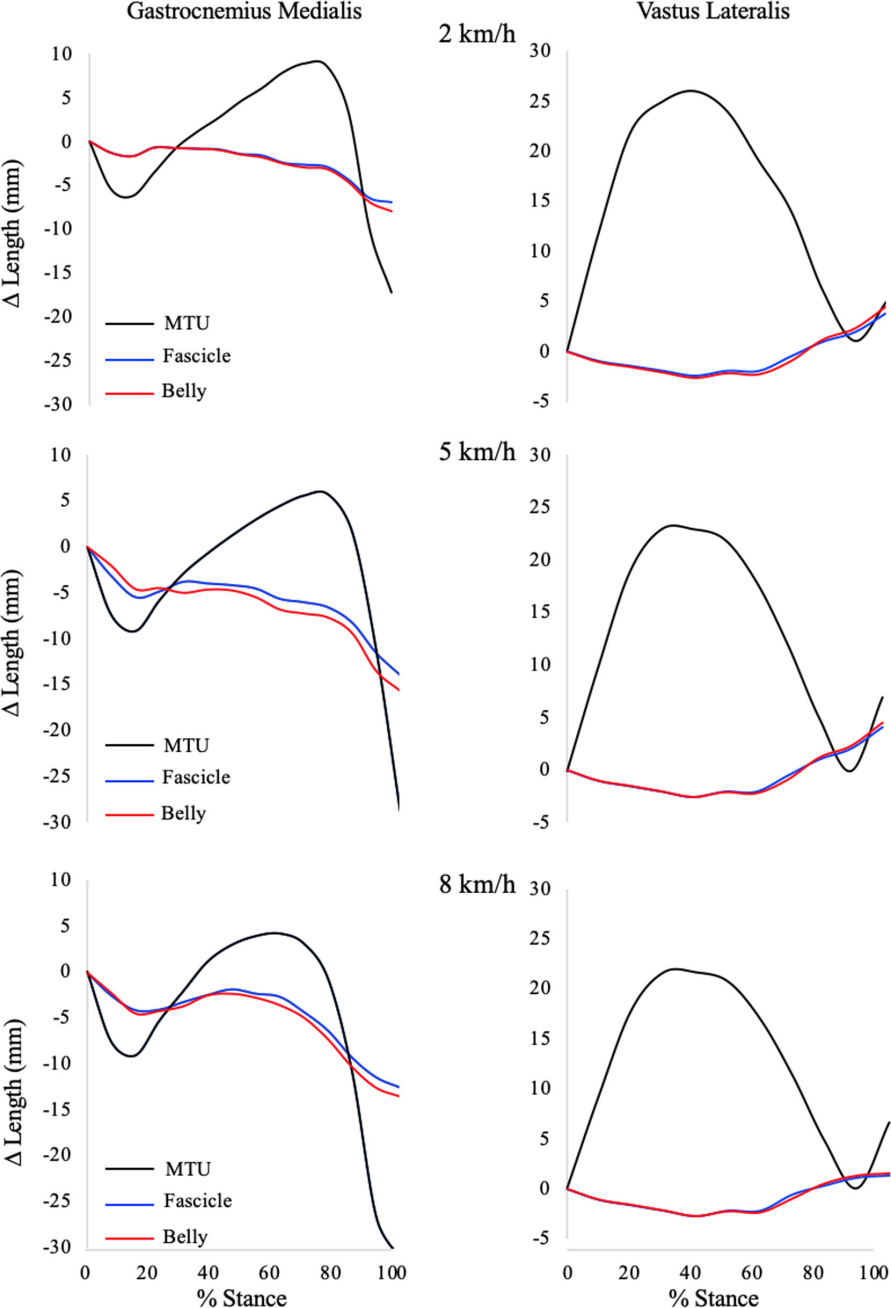
MTU (black), belly (red), and fascicle (blue) length behavior of gastrocnemius medialis (left panels) and vastus lateralis (right panels) during the stance phase while walking at three representative speeds (upper panels: 2 km.h^−1^; middle panels: 4 km.h^−1^; lower panels: 8 km.h^−1^) in a representative subject. Length values were “normalized” for the initial length at touch‐down (changes in length were calculated in respect to the initial length). Thus, negative and positive values denote shortening and lengthening in respect to the initial length, respectively

The MTU, muscle‐belly, and fascicles behaviors during the stance phase are reported for three representative walking speeds in Figure 1. The instantaneous values of MTU, fascicle, and muscle‐belly length were “normalized” to their initial length (at touch‐down) and are thus reported as changes in length throughout the stance phase (from touch‐town to take‐off). The instantaneous values of belly and tendon gearing (Gb and Gt, respectively), were calculated throughout the entire stance phase as the ratio between the instantaneous values of muscle‐belly and fascicle length changes (Gb = ∆muscle‐belly length/∆fascicles length) and MTU and belly length changes (Gt = ∆MTU length/∆muscle‐belly length). Finally, the average values of Gb and Gt (during the stance phase) were calculated and utilized in further analysis.

The EMG activity of VL, GM, TA, and BF was analyzed for ten stride phases. The raw EMG signal collected during walking was filtered with a band‐pass third‐order Butterworth filter at 20–450 Hz, and the root‐mean square (RMS) was calculated using a moving window of 25 ms throughout the entire stride. The cumulative activity per distance travelled (CMAPD) was calculated for each muscle as proposed by Carrier et al.^30^:

$$CMAPD=\frac{\mathrm{meanEMG}}{v}$$

Where mean EMG is the mean EMG amplitude during the ten analyzed strides and v is the walking speed (expressed in m·s^−1^). Total CAMPD (CMAPD_TOT_) was calculated as the sum of the CMAPD of all the investigate muscles.^35^

For the metabolic measurements, oxygen uptake ($\dot{V}$O_2_) at rest was subtracted from $\dot{V}$O_2_ during exercise, in order to obtain net oxygen consumption ($\dot{V}$O_2net_, expressed in mlO_2_·min^−1^·kg^−1^). Net energy cost (C_net_) was calculated as: $\dot{V}$O_2net_/v (where v is the treadmill speed, m·s^−1^) and expressed in J·m^−1^·kg^−1^ by using an energy equivalent (J·mlO_2_^−1^) that takes into account the respiratory exchange ratio.^36^

### 2.4 Statistical analysis

Values are presented as means ± SD. A repeated‐measures ANOVA with a Bonferroni adjustment was used to test for differences across all speeds in all the investigated variables. The calculation of the effects size (ES) was also included. ES was classified as trivial = <0.20, small = 0.20–0.50, moderate = 0.50–0.80 or large >0.80.

The correlations among C_net_, EMG activity (CMAPD_TOT_), belly gearing, and tendon gearing were first assessed with independent Pearson's correlation coefficient. Since the possibility of false positive correlations increases with the number of correlations performed, Pearson's product moment correlation *p* values were corrected for multiple tests using the Benjamini–Hochberg procedure^37^ with a false detection rate of 5% (significance was defined as adjusted *p* < 0.05).

We further conducted a multiple stepwise linear regression analysis to assess the magnitude of the effect of the analyzed variables on the energy cost of walking at all the investigated speeds. The predictive variables were checked for collinearity: when the variance inflation factors (VIF) were larger than 5 for any predictor variable, the variables were standardized (e.g., by subtracting the mean) before they were used in the model. Statistical analyses were conducted using R (v.31.1).

## 3 RESULTS

**FIGURE 2:**
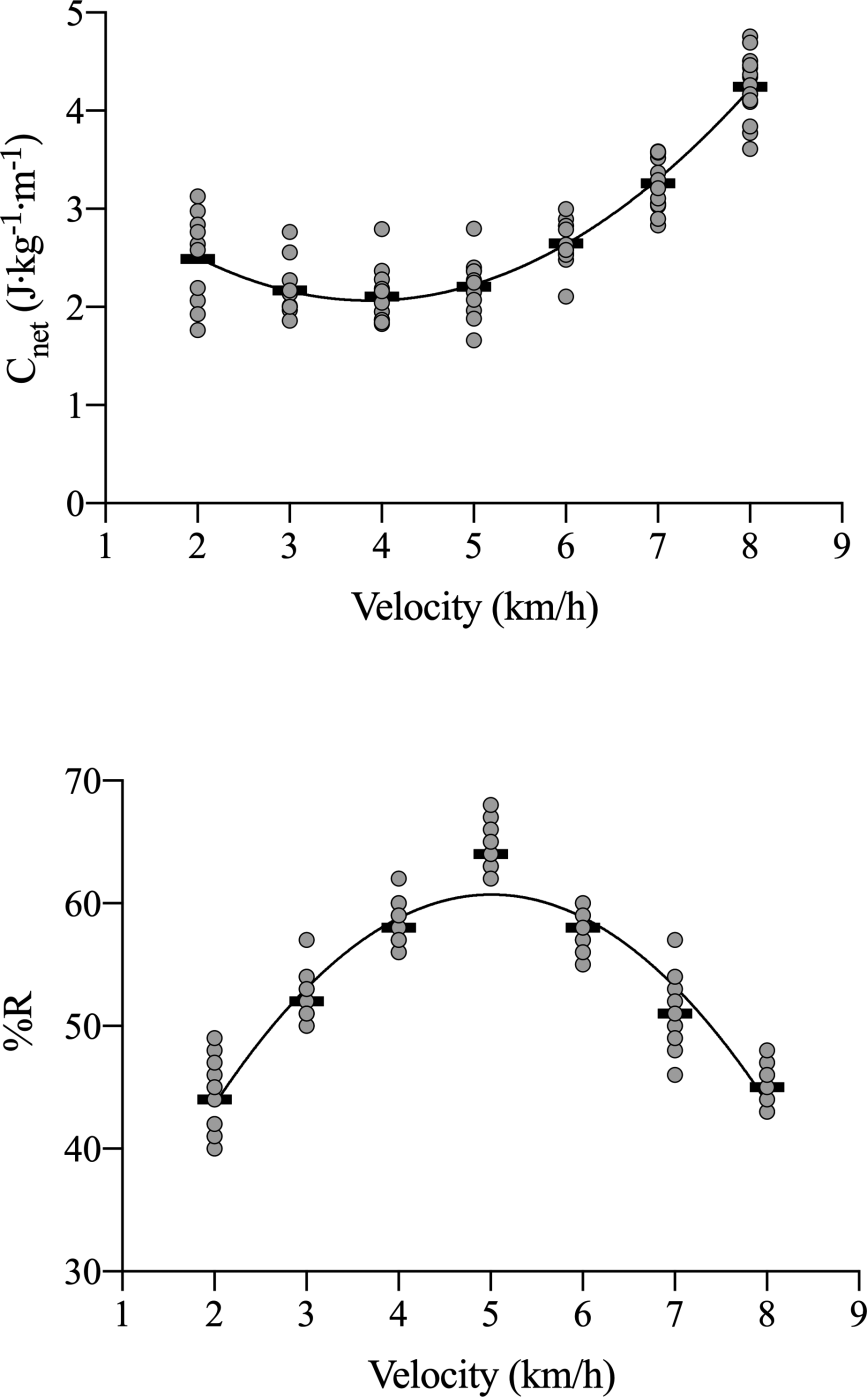
Net energy cost (C_net_, upper panel) and pendular energy recovery (%R, lower panel) as a function of walking speed. Black rectangles represent the mean values at each walking speed, while gray dots the individual subjects

Net energy cost (C_net_) and pendular energy recovery (%R) changed significantly with walking speed (*p* < 0.001, in both cases; ES = 0.83 and 0.85 for C_net_ and %R, respectively), whereas C_net_ showed a U‐shaped behavior with its minimum at about 4–5 km·h^−1^ (see Figure 2, upper panel), %R showed an opposite trend, reaching its maximum at 5 km·h^−1^ (see Figure 2, lower panel).

**FIGURE 3:**
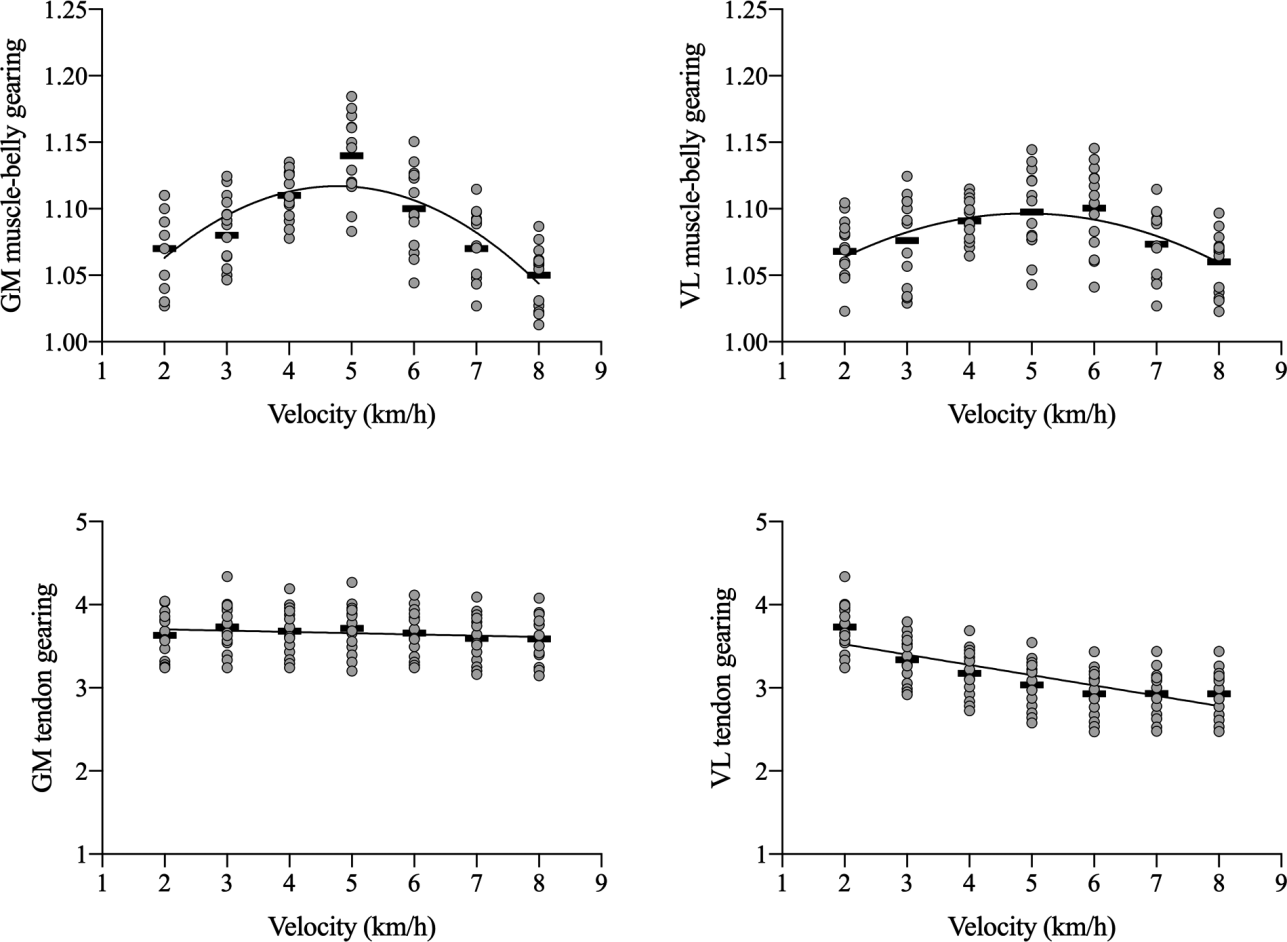
Vastus lateralis and gastrocnemius medialis muscle‐belly (upper panel) and tendon (lower panel) gearing as a function of walking speed (data refer to the stance phase). Black rectangles represent the mean values at each walking speed, while gray dots the individual subjects

The cumulative EMG activity per distance travelled (CMAPD) changed significantly with walking speed in all the investigated muscles (*p* < 0.001, in all cases) following a curvilinear (U‐shaped) function with a minimum at about 4 km·h^−1^ (see Figure 3). The effect sizes were: 0.81, 0.77, 0.80, and 0.71 for GM, TIB, VL, and BI, respectively.

Average length variation relative to the initial length at touch‐down for MTU, fascicle, and muscle‐belly are reported in Table 1. For GM, the changes in MTU, muscle‐belly, and fascicle length increased with speed (*p* < 0.001 for all parameters). For VL, the fascicle and muscle‐belly length changes increased as a function of walking speed (*p* < 0.01 for both parameters; ES = 0.57 and 0.58 for muscle‐belly and muscle fascicles, respectively), whereas the MTU length changes decreased as a function of it (*p* = 0.04, ES = 0.47).

**TABLE 1:** MTU, fascicle, and muscle‐belly length changes (average length variation relative to the initial length at touch‐down) of vastus lateralis and gastrocnemius medialis during the stance phase at the investigated walking speeds

Vastus lateralis				Gastrocnemius medialis		
Walking speed **(**km⋅h^−1^ **)**	∆ MTU length (mm)	∆ Fascicle length (mm)	∆ Muscle‐belly length (mm)	∆ MTU length (mm)	∆ Fascicle length (mm)	∆ Muscle‐belly length (mm)
*2*	9.81 ± 1.1	2.63 ± 0.5	2.45 ± 0.4	16.7 ± 2.9	4.31 ± 0.3	4.60 ± 0.5
*3*	9.20 ± 0.9	2.75 ± 0.4	2.55 ± 0.3	19.7 ± 3.6	4.92 ± 0.3	5.29 ± 0.4
*4*	9.16 ± 0.8	2.89 ± 0.4	2.65 ± 0.3	21.2 ± 2.7	5.23 ± 0.4	5.77 ± 0.6
*5*	9.09 ± 0.8	2.99 ± 0.3	2.73 ± 0.4	23.5 ± 2.6	5.61 ± 0.3	6.33 ± 0.5
*6*	8.95 ± 0.7	3.07 ± 0.5	2.80 ± 0.3	24.6 ± 3.4	6.10 ± 0.4	6.71 ± 0.8
*7*	8.79 ± 0.7	2.99 ± 0.5	2.82 ± 0.4	28.9 ± 3.9	6.77 ± 0.4	7.81 ± 0.7
*8*	8.81 ± 0.7	3.02 ± 0.4	2.85 ± 0.4	32.9 ± 4.0	7.36 ± 0.5	9.14 ± 0.6

Note

Data are means ± SD and allow to appreciate the uncoupling behavior between structures.

**FIGURE 4:**
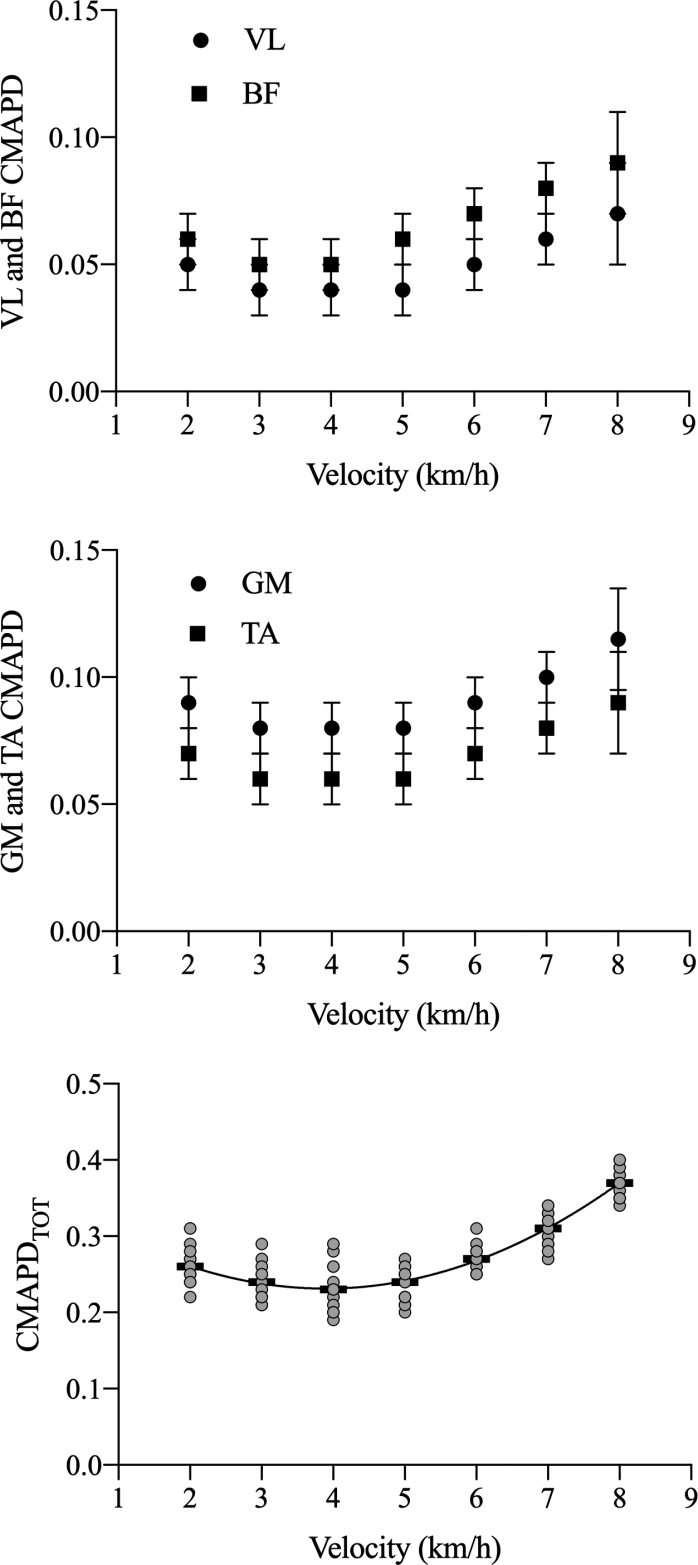
Cumulative EMG activity per unit distance (CMAPD) as a function of walking speed. Upper panel: vastus lateralis (VL), biceps femoris (BF); middle panel: gastrocnemius medialis (GM) and tibialis anterior (TA); lower panel: total CMAPD (i.e., sum of CMAPD for all muscles). In the upper and middle panel, values are reported as mean and standard deviation, while *n* the lower panel, black rectangles represent the mean values at each walking speed, while gray dots the individual subjects

At the slowest speed, tendon gearing was of about 3.5 in both MTUs; Gt decreased as a function of speed in VL (*p* = 0.045, ES = 0.49) and was unaffected by speed in GM (see Figure 4). Muscle‐belly gearing changed with speed both in GM and VL (*p* < 0.001, ES = 0.78 and *p* < 0.01, ES = 0.53, respectively) showing a parabolic trend with a maximum at about 5 km·h^−1^ (see Figure 4). Gb was similar in VL and GM at the slowest and fastest speeds (about 1.05), but its peak value was larger in GM than in VL (1.13 and 1.08, respectively).

### 3.1 Correlations between variables

**FIGURE 5:**
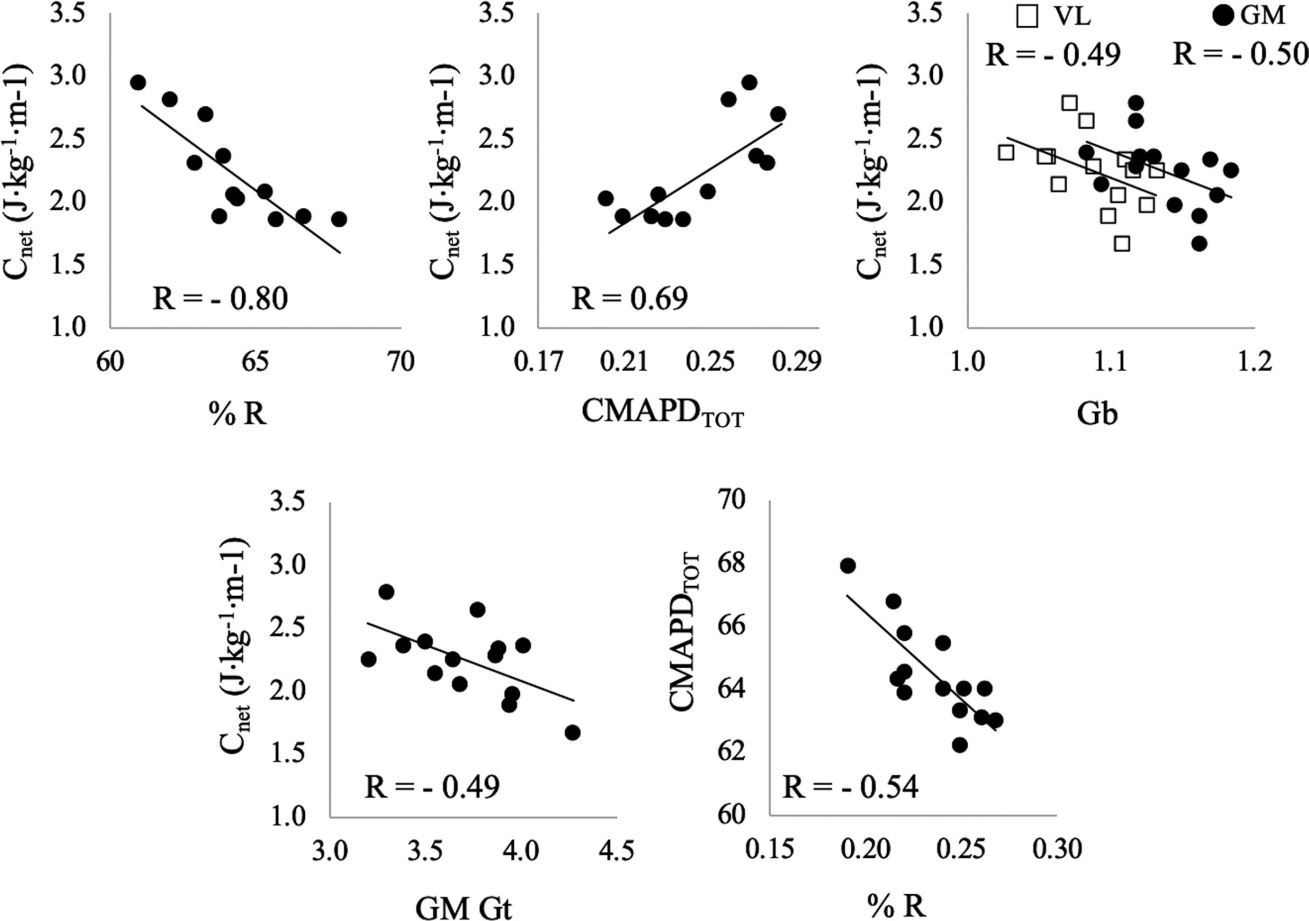
Example of correlations between net energy cost (C_net_) and (from top to bottom) energy recovery (%R), cumulative EMG activity per unit distance (CMAPD_TOT_), belly gearing and gastrocnemius medialis tendon gearing. Dots represent individual data points and refer to a speed of 5 km.h^−1^. Correlations were all significant. Further details about the correlation coefficients at each walking speed are reported in the main text

Correlations between variables and C_net_ were assessed using the corrected Pearson's product moment correlation coefficient (r) by means of the Benjamini–Hochberg procedure (see Statistical analysis). Examples of these correlations are reported in Figure 5 for a walking speed of 5 km.h^−1^ but similar trends were observed at all speeds.

Percentage recovery (%R) and total cumulative activity required per distance travelled (CMAPD_TOT_) were negatively (*p* < 0.001, %R) and positively (*p* < 0.01, CMAPD_TOT_) correlated with C_net_ at all the investigated speeds. Pearson's product moment correlation (r) ranged from −0.81 to −0.85 for %R and from 0.68 to 0.73 for CMAPD_TOT_.

Belly gearing of both muscles (GM and VL) was negatively correlated with C_net_ at all the investigated speeds (*p* < 0.05 in all cases): the higher the belly gearing the lower C_net_. Pearson's product moment correlation coefficient (r) ranged from −0.50 to −0.58 for GM and from −0.49 to −0.54 for VL, respectively.

Tendon gearing of GM was negatively correlated with C_net_ at all the investigated speeds (*p* < 0.05 in all cases). Pearson's product moment correlation (r) ranged from −0.46 to −0.49. VL tendon gearing was not correlated with C_net_.

GM and VL muscle‐belly and/or tendon gearing were not correlated with CMAPD of the respective muscle nor with CMAPD_TOT_. This suggest that the EMG activity of a muscle is uncoupled to its belly gearing. Finally, CMAPD_TOT_ and %R were negatively correlated (*p* < 0.05 at all the investigated speed) and the Pearson's product moment correlation (r) ranged from −0.48 to −0.54.

### 3.2 Multiple regression analysis

A multiple stepwise linear regression analysis was utilized to assess the magnitude of the influence of the analyzed variables on C_net_ at all the investigated speeds. The parameters included in the model were as follows: muscle‐belly and tendon gearing of VL and GM, %R, and CMAPD_TOT_. Since we were interested in understanding the strength of the influence that each variable has on C_net_ (and not to predict the energy cost), the statistical effects of each parameter (at all the investigated speeds) are reported in Table 2, whereas the resultant equations are not reported.

**TABLE 2:** Results of the multiple linear regression analysis. Data refer to standardized β coefficients and adjusted *p* values for the significant predictive variables in determining the energy cost of walking at all the investigated speeds

*Walking speed* (*km·h* ^−^ *^1^*)	*GM belly gearing*	*CMAPD_TOT_*	*%R*
*2*	Β = 20	β = 28	β = 46
	*p* = 0.043	*p* = 0.022	*p* = 0.002
*3*	β = 21	β = 32	β = 47
	*p* = 0.041	*p* = 0.023	*p* = 0.005
*4*	β = 23	β = 31	β = 44
	*p* = 0.039	*p* = 0.027	*p* = 0.002
*5*	β = 23	β = 32	β = 45
	*p* = 0.033	*p* = 0.028	*p* = 0.003
*6*	β = 22	β = 29	β = 49
	*p* = 0.038	*p* = 0.025	*p* = 0.003
*7*	β = 23	β = 30	β = 44
	*p* = 0.044	*p* = 0.027	*p* = 0.002
*8*	β = 24	β = 30	β = 45
	*p* = 0.045	*p* = 0.026	*p* = 0.002

Abbreviations: %R: pendular energy recovery; *CMAPD_TOT_*: cumulative EMG activity per unit distance; GM: gastrocnemius medialis.

GM and VL tendon gearing and VL belly gearing were excluded by the model at all the investigated speeds. Considering the standardized β coefficients, the model indicates that %R has the greatest influence on C_net,_ followed by CMAPD_TOT_ and GM belly gearing.

## 4 DISCUSSION

By analyzing whole‐body kinematics and muscle‐tendon behavior of the vastus lateralis and gastrocnemius medialis during walking at different speeds, we investigated the influence of two functionally different MTUs on the energy cost of walking.

In agreement with our hypotheses, we found that the ability to save mechanical energy by using a pendulum‐like mechanism (i.e., %R) and the EMG activity of the recruited muscles (i.e., per distance travelled: CMAPD_TOT_) are correlated to the energy cost (metabolic expenditure per distance travelled) of walking. These parameters are negatively correlated with each other since muscle activity can be reduced when the pendular exchange is maximized. Therefore, the parabolic trend of energy recovery and the U‐shape behavior of the EMG activity (of the analyzed muscles), help to explain the U‐shape behavior of the Cnet vs. velocity relationship in walking.

In addition, we provided novel evidence of a further optimization phenomenon at the muscles level during walking. Belly gearing of both muscles (VL and GM) showed, indeed, a parabolic trend reaching a maximum at about 5 km.h^−1^, near to the minimum of the C_net_ vs. velocity relationship (4–5 km.h^−1^). VL and GM belly gearing were negatively correlated with C_net_. Thus, a neuromuscular system able to operate with higher values of belly gearing could potentially reduce the energy cost of walking. However, VL belly gearing was excluded by the multiple linear regression model indicating that only the GM muscles mechanical behavior (i.e., belly gearing) could affect C_net_. It is worth noting that the “positive” influence of muscles mechanical behavior (belly gearing) is not related to a reduction of the EMG activity since no significant correlation was observed between these variables.

Finally, as expected, tendon gearing plays only a marginal role in determining the energy cost of walking. Even if GM tendon gearing was negatively correlated with C_net_ at all the investigated speeds, tendon gearing of both MTUs was excluded by the multiple linear regression model, suggesting a negligible role in determining C_net_ compared with the other variables.

### 4.1 Tendon gearing

Ultrasound data have provided evidence of the importance of the elastic components during walking.^8^, ^9^, ^13^, ^15^, ^16^, ^17^, ^38^ For example, Fukunaga et al.^13^ and Bohm et al.^38^showed that during the stance phase of walking, GM and VL MTUs exhibit a notable displacement, whereas muscle fascicles operate with significantly smaller length changes. This uncoupling behavior between MTU and fascicle length changes demonstrates that the main length changes of the MTU are primarily associated with those of the series‐elastic elements. These results are in agreement with those reported in the current study that indicates that the displacement of the elastic structures was approximately 3–4 times larger than that of the fascicles and of the muscle‐belly (Gt of about 3.7 and 3 for GM and VL, respectively). Tendon elasticity, by decoupling muscle length changes from MTU length changes, allows the muscle fascicles to operate closer to their optimal fascicle length and this mechanism, in combination with the tendon's capability of storing and releasing elastic strain energy, allows for a reduction in the metabolic demand in energy‐saving tasks like walking.^20^

However, VL and GM tendon gearing did not show an optimization phenomenon: VL tendon gearing decreased as a function of walking speed, whereas GM tendon gearing was unaffected by it. The differences between GM and VL tendon gearing behavior as a function of speed, could be explained by the different functional role of the two MTUs. While the quadriceps MTUs mainly decelerate and support the body throughout the stance phase,^38^, ^39^ the plantar flexors muscles contribute to the acceleration of the BCoM during the second part of the stance phase.^17^, ^39^ Therefore, the energy stored by the Achilles tendon contributes to a greater proportion of the MTU propulsive mechanical work, whereas the energy stored by the VL elastic structures does not contribute to positive work generation and a simple energy transfer within the MTU could allow for a minimization of the active muscle volume.

The decreases in VL tendon gearing could be related with knee joint kinematics. Knee joint range of motion increases as a function of walking speed^39^ and the increase in knee joint flexion could reduce the force acting along the patellar tendon, thereby reducing the elastic displacement created by the external forces and thus tendon gearing.

Regarding GM tendon gearing, Farris & Sawicki^17^ pointed out that the mechanical power released by the series‐elastic component of the gastrocnemius medialis remains similar during walking at increasing speeds, explaining approximately 45% of the mechanical power generated by the MTU,^17^ and this could explain why Gt did not change with walking speed and the exclusion of GM tendon gearing by the regression model. Therefore, even if tendon gearing could play an important role in energy‐saving tasks such as running or bouncing,^40^ it could not explain the changes in energy cost of walking when walking speed increases.

### 4.2 Belly gearing

A novel finding of this study regards muscle‐belly gearing behavior: VL and GM belly gearing exhibited an optimization phenomenon around the optimal walking speed (where C_net_ is minimized). This behavior could be related to changes in muscle force. As reported in different experimental conditions,^21^, ^23^, ^41^ Gb is high during low‐force and high‐velocity contractions and decreases when the force exerted by the muscle increases.^21^ The changes in Gb are mediated by 3D muscle shape changes, that are probably impeded when the pressure on the connective tissue elements is elevated (e.g., when intramuscular force is elevated it tends to compress the muscle).^21^ Advances in research devices measuring muscle force (e.g., tapping tendon) and 3D muscle shape changes (e.g., 3D ultrasound analysis) are expected to provide further insight into the underpinning mechanism of belly gearing during locomotion.

The first observations of Azizi et al.^21^ using in situ animal preparation were confirmed by recent in vivo studies: during cycling,^41^ and during explosive dynamic contractions,^23^ belly gearing was shown to decrease when muscle force increases. To the best of our knowledge, only one study investigated muscle force in vivo during walking at increasing speeds. Finni et al.^42^ using optic fiber technique showed that the Achilles tendon force, as well as the rate of force along the Achilles tendon line of action, increases as a function of walking speed (from 1.2 to 1.8 m.s^−1^). Thus, in agreement with data reported by Finni and co‐workers, our data suggest that above the optimal walking speed belly gearing decreases because the force applied by the plantar flexor muscles increases, and this is in accordance with the previously mentioned studies.^21^, ^23^, ^41^ Unfortunately, Finni and co‐workers did not investigate walking speeds lower than the optimal (e.g., in the descending limb of the C_net_ vs. velocity relationship). However, based on the metabolic counterpart, it is possible to hypothesize that the muscle force generated to stabilize and propel the body during the stance phase is minimized at the optimal walking speed, and increases at larger or lower speeds: “walking like an inverted pendulum” indeed reduces the muscle force demands during single support, even if it requires mechanical work to redirect the body center of mass in the transition between steps.^5^, ^6^

Thus, a lower level of force (at the optimal walking speed) could promote the uncoupling between fascicle and belly shortening velocity, allowing the muscle fascicles to work at a high F‐V potential (e.g., operating at slower velocities),^22^, ^43^ reducing the energy requirements at this speed. Otherwise, when the neuromuscular system is not able to promote high values of belly gearing, the fascicle length changes would be higher, increasing the cross‐bridge turnover and the energy demands for muscle contraction.^18^, ^19^ Our results partially support these hypotheses: belly gearing was negatively related with the energy cost of walking at all the investigated speed (the higher the uncoupling behavior between whole‐muscle structures and muscle fascicles the lower the energy cost of walking). Therefore, for the same walking speed, neuromuscular system able to operate with higher values of belly gearing allow the muscle fascicle to operate under more favorable portion of the F‐L and F‐V potential, thereby reducing the metabolic demand.

As pointed out by Azizi et al,^21^ the mismatch between fiber velocity and muscle velocity is not mediated by nervous control over shape changes, but rather, by the interaction between contractile and connective tissues. These results were recently confirmed by Dick & Wakeling^41^ and Monte et al.^23^ who observed a significant negative correlation between muscle force and belly gearing, but no relationship between EMG activity and muscle force, suggesting an uncoupling behavior between muscle‐belly gearing and EMG activity. Our data confirmed their results, indicating that belly gearing behavior could not be explained by EMG behavior (e.g., CMAPD, in this study) in accordance with previous literature.^21^, ^41^, ^44^

The advantage of a higher belly gearing on the energy cost should thus be attributed to its role in determining muscle work. For a given amount of muscle shortening, the higher the belly gearing the lower the fascicles shortening compared with that of the muscle. As a consequence, the mechanical work produced by the muscle fascicles is expected to be lower the higher the belly gearing, thereby reducing the energy cost of contraction. Indeed, GM belly gearing was accepted into the regression model as a significant determinant of C_net_. This was not the case for VL belly gearing, and this could be explained by the different behavior of these muscles.

The GM muscle fascicles shorten during the entire stance phase, indicating a continuous mechanical work production, whereas the VL fascicles remain quasi‐isometric during the stance phase, thus minimizing mechanical work production. As reported by Bohm et al. (2018), VL operates at high force‐length and force‐velocity potential during the stance phase of walking and this minimizes the energy demands of this muscle, which is energetically expensive due to its long fascicle length. Leg muscle modeling studies support the idea of GM being more metabolically demanding compared with other leg muscles,^45^ and previously published data showed that GM exhibits speed‐dependent decreases in operating length, and faster shortening during the push off phase in comparison with soleus.^46^ Our data, in accordance with these studies, suggest that the uncoupling behavior between the GM muscle‐belly and its muscle fascicle could play a role in reducing the mechanical work provided by GM muscle fascicles, reducing C_net_. On the contrary, the influence of VL belly gearing seems not sizeable enough, due to the small amount of fascicle shortening exhibited by the VL active components.

In this study, we analyzed the influence of VL and GM muscle‐tendon behavior on the energy cost of walking. Other propulsive muscles, with larger PCSA and volume (e.g., soleus and biceps femoris) are expected to play a significant role in determining metabolic energy expenditure during walking. We decided to investigate the vastus lateralis and gastrocnemius medialis MTUs since they well represent the contribution of the quadriceps and plantar flexors muscle together^39^ and because it was observed (modeling studies) that, in the triceps surae, GM is the more metabolically demanding muscle during walking.^45^ Future studies on other lower limb MTUs could shed further light on the determinants of the cost of transport in human walking.

Since belly gearing is strongly affected by the changes in muscle thickness, it is important to note that securing the ultrasound transducer onto the leg will inherently constrain movement of the superficial surface of both muscles (e.g., VL and GM). This is an unavoidable problem, given that the transducer must be secure to enable capture of the required images. In order to reduce this bias, we applied a soft gel tap between the ultrasound probe and the skin. As a result, the muscles had the capacity to expand radially. In fact, in both muscles, we noted vertical displacement of the ultrasound probe, highlighted by the kinematic of the marker placed on the involucrum, suggesting the muscles capability to change in thickness. However, the impact of this procedure could not be quantified.

## 5 PERSPECTIVE

Our data provide new in vivo evidence that belly gearing shows an optimum during walking at about 5 km.h^−1^, near to the optimal walking speed and to the minimum of the C_net_ vs. velocity relationship. The mechanical behavior, and in particular the uncoupling behavior of muscle's active components of the gastrocnemius medialis (but not vastus lateralis) seem to play a role throughout a reduction of the mechanical work provided by the muscle fascicles.

These results provide new insight on the determinants of the energy cost of walking at different speeds, suggesting that increases in the energy cost of walking could be related to (i) an impairment of the pendular energy exchange mechanism; (ii) the uncoupling behavior of the contractile component, and/or a combination thereof.

## CONFLICT OF INTERESTS

We declare that we have no competing interests.

## AUTHOR CONTRIBUTIONS

AM, PT, FN, LPA, and PZ designed research. AM, PT, FN, and BB performed research. AM and PT analyzed data. AM and PZ drafted the manuscript. PT, FN, BB, and LPA made important intellectual contributions during revision.

## ETHICAL APPROVAL

The local ethical committee approved the experimental protocol (protocol number 2020‐UNVRCLE‐161 0142370), and all subjects gave their written informed consent.

## Data Availability

The data that support the findings of this study are available from the corresponding author upon reasonable request.
